# Clinical Characteristics and Cardiac Rehabilitation Outcomes During the Perioperative Period After MIDCAB and OPCAB Surgery: A Comparative Study

**DOI:** 10.3390/jcdd12090331

**Published:** 2025-08-28

**Authors:** Yao Wu, Bao Ren, Jing Li, Liqun Chi, Ping Li, Jiahui Wu

**Affiliations:** 1 Department of Cardiac Rehabilitation Center, Beijing Anzhen Hospital, Capital Medical University, Beijing 100029, China; wylvyxc@163.com (Y.W.); renbao162@163.com (B.R.); 2 Department of Minimally Invasive Surgery Center, Beijing Anzhen Hospital, Capital Medical University, Beijing 100029, China; 15910840422@163.com (J.L.); 13911872318@163.com (L.C.); 13520112185@163.com (P.L.)

**Keywords:** 6MWD, cardiac rehabilitation, HRV, MIDCAB, OPCAB, perioperative, pulmonary function

## Abstract

Background: Minimally invasive direct coronary artery bypass (MIDCAB) surgery offers advantages over off-pump coronary artery bypass (OPCAB), including reduced trauma and faster recovery. However, differences in perioperative cardiac rehabilitation (CR) outcomes between MIDCAB and OPCAB remain unclear. This study compared perioperative clinical characteristics, surgical features, and CR outcomes in patients undergoing MIDCAB versus OPCAB. Methods: This retrospective cohort analysis included 304 patients (31.2% MIDCAB, 68.8% OPCAB) who participated in a CR program, including the 6-min walk test (6MWT), from November 2023 to December 2024. Results: MIDCAB patients had shorter surgery times, fewer grafted vessels, shorter ventilator-assisted time, less total intraoperative fluid, less bleeding, and shorter postoperative hospital stays (all *p* < 0.05). After cardiac rehabilitation, MIDCAB patients showed shorter time to 6MWT, longer six-minute walk distance (6MWD) (200 ± 125 vs. 178 ± 125 m), higher 6MWD relative to predicted values, and greater metabolic equivalents (all *p* < 0.05). The median LVEF of MIDCAB patients was the same as that of OPCAB patients (*p* < 0.05). Conclusions: Our study demonstrates that MIDCAB patients exhibit superior exercise capacity following cardiac rehabilitation.

## 1. Introduction

Coronary artery disease (CAD) is currently the third leading cause of death globally [1,2]. Coronary artery bypass grafting (CABG) has been recognized as a well-established surgical procedure used to treat significant CAD [3]. With advancements in medical technology, minimally invasive direct coronary artery bypass (MIDCAB) has garnered increasing attention due to its minimal invasiveness and rapid recovery [4,5]. Compared to traditional off-pump coronary artery bypass (OPCAB), MIDCAB shows a clear advantage in reducing surgical trauma and facilitating faster rapid postoperative recovery. However, more clinical data are needed to further validate these benefits [6,7].

The American Association of Cardiovascular and Pulmonary Rehabilitation (AACVPR) recommends that cardiac rehabilitation (CR) for patients with CAD should be divided into three distinct phases: phase I rehabilitation (in-hospital rehabilitation), phase II rehabilitation (out-of-hospital early rehabilitation/outpatient rehabilitation), and phase III rehabilitation (out-of-hospital long-term rehabilitation) [8]. The perioperative CR program, which includes preoperative prehabilitation and postoperative phase I rehabilitation, is a crucial component in the recovery of patients undergoing CABG, aiming to help patients restore cardiac function, improve quality of life, and reduce the risk of disease recurrence through comprehensive interventions [9]. Several large-scale trials and meta-analysis have demonstrated that CR improves long-term survival in CABG patients [10,11,12]. The six-minute walk test (6MWT), a common outcome measurement in CR, has been found to be responsive to changes in clinical status following CR, making it a useful tool for assessing the effectiveness of perioperative CR [13]. Recently, Carolin Steinmetz et al. reported improvements in functional capacity and quality of life in perioperative CABG patients with rehabilitation, but they did not directly compare rehabilitation outcomes between OPCAB and MIDCAB [14].

This study aims to compare the perioperative clinical characteristics, surgical features, exercise capacity (as measured by the 6MWT), left ventricular function (assessed using ultrasonic cardiogram, UCG), autonomic function (evaluated by heart rate variability, HRV), and pulmonary function outcomes in patients recovering from both MIDCABG and traditional OPCABG following the phase Ⅰ CR program. This comparison is of significant importance for optimizing surgical techniques, enhancing postoperative recovery outcomes, and reducing the risk of disease recurrence.

## 2. Materials and Methods

### 2.1. Study Population

This is a retrospective cohort study involving 304 patients with CAD, with a median age of 63 years (range 38–80), including 237 males and 67 females. The study was conducted from 22 November 2023 to 9 December 2024 and included patients who underwent either isolated OPCAB (n = 209) or isolated MIDCAB (n = 95) in the Minimally Invasive Surgery Center of Beijing Anzhen Hospital. Patients in whom a planned MIDCAB procedure was converted to sternotomy and OPCAB were excluded. Patients requiring concomitant procedures were excluded. Prior to surgery, all participants underwent prehabilitation, which included abdominal breathing and cough training. Upon returning to the ward from the intensive care unit (ICU), they initiated the phase Ⅰ CR program, both in bed and at the bedside, under the guidance of therapists. The details of the standardized phase Ⅰ CR program for CABG patients who have been reported elsewhere [15]. This program consisted of respiratory and cough training, active upper and lower limb movement, transfer training, standing training and walking exercises. Subsequently, they performed the 6MWT following the phase Ⅰ CR program and prior to hospital discharge, at a median of 5 ± 2 days after surgery. The 6MWT and the pulmonary function were both performed only postoperatively. Patients’ medical records were reviewed and assessed by two independent researchers. The study was conducted in accordance with the Declaration of Helsinki, and the protocol was approved by the Ethics Committee of hospital and informed consent was obtained from all study participants (KS2024024) on 13 March 2024.

### 2.2. Surgical Procedures and Revascularization

Surgical approach (MIDCAB vs. OPCAB) was determined by the operating surgeon based on coronary anatomy, comorbidities, and institutional protocols. The MIDCAB was performed via left anterior thoracotomy for left anterior descending artery revascularization using the left internal mammary artery. The OPCAB was performed via median sternotomy for revascularization. The MIDCAB group usually had single or two-vessel disease with the number of grafted vessels of 2 ± 2. The OPCAB group had three-vessel disease with the number of grafted vessels of 3 ± 1. The term ‘grafted vessels’ refers to the number of distal anastomoses performed. Completeness of revascularization was confirmed intraoperatively by the surgical team using graft patency testing and angiography. All MIDCAB patients received complete myocardial revascularization of targeted vessels.

### 2.3. 6MWT

In accordance with the guidelines of the American Thoracic Society, the 6MWT was performed using a smart six minute walking test and analysis system (Wocaring Medical Equipment Co., Ltd., Wuhan, China) [16]. Patients wore intelligent monitoring devices (YK-2020A, Wocaring Medical Equipment Co., Ltd., Wuhan, China) and were instructed to walk the greatest possible distance along a 25 m straight, flat hospital corridor within six minutes [17]. The test was immediately discontinued if peripheral oxygen saturation (SpO_2_) < 85% or if the patient experienced exhaustion, angina, severe dyspnea, dizziness, diaphoresis, or intolerable leg cramps. The results of the 6MWT were given as absolute value in meters and as a percentage of the predicted value, taking into account anthropometric variables (age, sex, weight and height) according to the reference equation proposed by Enright and Scherril in healthy subjects [18]. The metabolic equivalent (MET) of the patients was automatically calculated based on the 6MWT results and recorded. Oral informed consent was obtained from all participants.

### 2.4. Pulmonary Function

Pulmonary function was assessed using specific software (version 2.0, LFD-168X, Wocaring Medical Equipment Co., Ltd.) before and after the 6MWT. Basic information, including gender, age, height and weight, was collected for these patients. Measurements were taken for the percentage of predicted values of forced vital capacity (FVC), forced expiratory volume in the first second (FEV1), peak expiratory flow (PEF), and FEV1/FVC ratio, as well as forced expiratory flow at 25% (FEF25), 50% (FEF50), and 75% (FEF75) and between 25% and 75% (FEF25–75) of vital capacity.

### 2.5. HRV

Throughout the 6MWT, HRV was assessed using dynamic cardiopulmonary function data analysis software (version 2.0, Wocaring Medical Equipment Co., Ltd.). The cardiac monitor captured normal-to-normal RR intervals (NN) and conducted HRV analysis through time-domain, frequency-domain, and nonlinear measurements. Measures in the time-domain analysis were as follows: normal-to-normal variability in the geometric range (NNVGR); standard deviation of all NN intervals (SDNN), which reflects the circadian heart rhythm; root mean square of the difference between the coupling intervals of adjacent NN intervals (RMSSD), which correlates with parasympathetic nervous system activity; standard deviation of successive differences (SDSD); number of interval differences in successive NN intervals greater than 50 ms (NN50), which primarily represents vagal activity; and the proportion of NN intervals differing by more than 50 ms (PNN50). In the frequency-domain analysis, total power [TP, (≤0.4 Hz)], which reflects overall autonomic activity, was measured and subdivided into the very-low-frequency range [VLF, (0.003–0.04 Hz)], the low-frequency range [LF, (0.04–0.15 Hz)], which is predominantly a marker of sympathetic nervous system activity, and the high-frequency range [HF, (0.15–0.40 Hz)], which reflects the modulation of parasympathetic nervous system efferent activity by ventilation. The LF/HF ratio, which reflects autonomic balance, was also measured in the frequency domain; a higher LF/HF ratio indicates a predominance of the sympathetic nervous system. Nonlinear measures were evaluated using a Poincaré plot, including standard deviation perpendicular to the line-of-identity (SD1), which describes short-term variability (representing parasympathetic modulation); standard deviation along the line-of-identity (SD2), which describes long-term variability (representing overall cardiac autonomic activity); and the SD2/SD1 ratio.

### 2.6. UCG

All patients underwent Doppler echocardiographic evaluations in accordance with the guidelines of the American Society of Echocardiography, both at admission and prior to discharge. Left atrial diameter (LAD), left ventricular end-diastolic diameter (LVEDD), and left ventricular ejection fraction (LVEF) were measured.

### 2.7. Statistical Analysis

All statistical analyses were conducted using Statistical Product and Service Solutions (version 26.0, SPSS Inc., Chicago, IL, USA). The Shapiro–Wilk test was applied to verify the hypothesis of normality of the variables. For normally distributed continuous variables, comparisons between groups were made using the independent Student’s *t*-test. Non-normally distributed continuous variables were analyzed with the Mann–Whitney U test, while categorical variables were evaluated using the chi-squared test for between-group differences. The Repeated-Measures ANOVA was utilized to compare pulmonary function parameters in the MIDCAB and OPCAB groups before and after the 6MWT. Results are presented as median ± interquartile range (IQR) or frequency (percentage), with statistical significance set at *p* < 0.05.

## 3. Results

### 3.1. Perioperative Clinical Characteristics

In total, 306 CABG patients were included in this study. The MIDCAB group comprised 95 patients (31.2%), while the OPCAB group included 209 patients (68.8%). The study population consisted of 237 men (78.0%) and 67 women (22.0%). The median age of the patients was 63 ± 12 years, and the median time from surgery to the 6MWT was 5 ± 2 days.

Table 1 summarizes the demographic and baseline clinical characteristics of the two patient groups. The percentage of males in the MIDCAB group was higher than that in the OPCAB group [81 (85.3%) vs. 156 (74.6%), χ^2^ = 4.289, *p* = 0.038]. The proportion of patients with mitral insufficiency was greater in the OPCAB group than in the MIDCAB group [34 (16.3%) vs. 7 (7.4%), χ^2^ = 4.433, *p* = 0.035], and it was the same in prior myocardial infarction [23 (11.0%) vs. 2 (2.1%), χ^2^ = 6.854, *p* = 0.009]. There were no significant differences in other characteristics between the two groups.

### 3.2. Surgical Features

There was no significant difference in ICU length of stay after surgery (*p* > 0.05). The MIDCAB group usually had single- or two-vessel disease, whereas the OPCAB group had three-vessel disease. However, in the OPCAB group, several variables were higher compared to the MIDCAB group: total surgery time [4.03 ± 1.66 vs. 4.25 ± 1.33 h, Z = −2.418, *p* = 0.016], number of grafted vessels [2 ± 2 vs. 3 ± 1, Z = −7.821, *p* < 0.001], ventilator-assisted time [4.18 ± 1.75 vs. 4.40 ± 2.29 h, Z = −2.333, *p* = 0.020], total intraoperative fluids [2000 ± 750 vs. 2500 ± 1000 mL, Z = −4.787, *p* < 0.001], intraoperative bleeding [400 ± 200 vs. 600 ± 300 mL, Z = −8.948, *p* < 0.001], and postoperative hospital stays [5 ± 1 vs. 6 ± 1 days, Z = −5.379, *p* < 0.001] (Table 2).

### 3.3. Perioperative Cardiac Rehabilitation Outcomes

Table 3 summarizes the exercise capacity (as measured by the 6MWT), autonomic function (evaluated by HRV), and left ventricular function (assessed using UCG) of CABG patients. MIDCAB patients exhibited a shorter mean postoperative time to the 6MWT [4.62 ± 1.37 vs. 5.33 ± 1.54 days, Z = −3.501, *p* < 0.001], longer median 6MWD [200 ± 125 vs. 178 ± 125 m, Z = −2.773, *p* = 0.006], higher median 6MWD as a percentage of predicted value [35 ± 23 vs. 33 ± 20%, Z = −2.506, *p* = 0.012], and greater median METs [2.70 ± 0.80 vs. 2.60 ± 0.80, Z = −2.823, *p* = 0.005]. The median LVEF of MIDCAB patients was the same as that of OPCAB patients [60 ± 5 vs. 60 ± 7, Z = −2.886, *p* = 0.004]. There were no significant differences in all HRV measures, LAD, and LVEDD between the two groups (all *p* > 0.05).

Table 4 presents the changes in pulmonary function before and after the 6MWT in both the MIDCAB and OPCAB groups. The interaction effects of all variables were not significant (*p* > 0.05), which means that the improvement in lung function by 6MWT did not differ between the two groups. That is, the changes in lung function before and after 6MWT were similar in the MIDCAB and OPCAB groups. In terms of time effect, the percentage of predicted values for FEV1, PEF, FEV1/FVC, FEF25, FEF50, FEF75, and FEF25–75 all showed significant improvement after the 6MWT, regardless of the type of surgery (all *p* < 0.05). In addition, the between-group effects for all variables were not significant (*p* > 0.05), indicating that there was no statistical difference between the MIDCAB and OPCAB groups in lung function indicators. These findings demonstrate that the 6MWT performed early in the postoperative period significantly improved pulmonary function (except for FVC) in patients undergoing either MIDCAB or OPCAB, and this improvement did not differ between the two surgical modalities.

## 4. Discussion

To the best of our knowledge, this is the first study to compare the outcomes of a perioperative CR program between patients undergoing MIDCAB and traditional OPCAB. A total of 304 CABG patients who participated in a perioperative CR program and completed the 6MWT from 22 November 2023 to 9 December 2024 were investigated. Our findings showed that MIDCAB patients had shorter total surgery time, fewer grafted vessels, shorter ventilator-assisted time, less total fluid infusion and bleeding, and shorter postoperative hospital stays compared to OPCAB patients. Following the phase Ⅰ CR program, patients who underwent MIDCAB surgery experienced a shorter average time from surgery to the 6MWT, longer 6MWD, higher 6MWD as a percentage of predicted values, and greater METs.

Previous studies have reported that MIDCAB patients have shorter ICU stay (0.78 ± 1.7 vs. 1.91 ± 1.01 days) and shorter hospital stay (6.78 ± 2.4 vs. 8.01 ± 2.5 days) compared to OPCAB patients (*p* < 0.05) [19]. Similarly, MIDCAB patients had fewer transfusions (0.93  ± 1.83 vs. 1.61 ± 2.52 units, *p*  <  0.001) and shorter mechanical ventilation time (7.6  ±  4.7 vs. 12.1  ±  26.4 h, *p*  =  0.005) [20]. In a study by Xu et al., the number of distal anastomotic stomas, operation time, blood transfusion volume, ventilator use time, ICU stay, and hospital stay were all significantly lower in the MIDCAB group compared to the traditional OPCAB group (*p* < 0.05) [21]. These findings are consistent with our study, highlighting the advantages of MIDCAB, such as small trauma, reduced bleeding, and rapid postoperative recovery. ICU length of stay, however, showed no significant difference between the two groups; this is in contrast to the findings of aforementioned studies, which could be due to the decision making of surgeons.

Additionally, the physical activity level depicts the energy expenditure resulting from activity, adjusted for an individual’s basic metabolic rate [22]. After the phase Ⅰ CR program, we observed that the median 6MWD was 200 ± 125 m in MIDCAB patients and 178 ± 125 m in OPCAB patients (*p* < 0.05). The mean time from surgery to the 6MWT was 4.62 ± 1.37 days for MIDCAB patients and 5.33 ± 1.54 days for OPCAB patients (*p* < 0.05). Our data demonstrate that MIDCAB patients exhibited higher physical activity level compared to OPCAB patients. To the best of our knowledge, there are no published reports on the effects of perioperative CR program specifically for MIDCAB groups using the 6MWT. Therefore, we cannot compare our findings directly with the existing literature, but our results may help to address this issue better. In Opasich et al.’s study, patients who underwent preoperative and postoperative cardiopulmonary rehabilitation had a mean 6MWD of 299 ± 72 m within 5.9 ± 1.1 days after surgery [23]. However, the 6MWD was shorter in our trial, which might be attributed to the shorter time from surgery to the 6MWT. Md Shajedur Rahman Shawon et al. reported that the mean 6MWD in patients undergoing a phase I CR program after cardiothoracic surgery was 359 ± 99 m at 8.5 ± 3.2 days postoperatively, and patients undergoing combined CABG and valve surgery covered significantly shorter distances during the 6MWT compared to those undergoing isolated CABG or valve-only procedures [24]. We found that smaller surgical incisions may be associated with longer 6MWD, suggesting that the 6MWD is influenced by the type and complexity of the cardiac surgery procedure [25,26]. Furthermore, the mean 6MWD was 303 ± 84 m within 15 days (9 ± 2 days) after CABG surgery. In the group of patients who repeated the 6MWT after a phase II CR program, the distance walked significantly increased [26]. This evidence suggests that preoperative prehabilitation, postoperative phase I CR, and phase II CR following hospital discharge can enhance the physical functional capacity of CABG patients to varying degrees. Moreover, the earlier the rehabilitation begins, the faster the recovery of physical capacity may be [23,26,27].

Previous studies reported a decrease in traditional HRV parameters immediately following surgery, with the lowest values typically observed between 3 and 6 days post-CABG [28,29]. While perioperative CR programs have been shown to lead to positive clinical outcomes, particularly in the MIDCAB group, it remains unclear whether the HRV performance following such programs differs between MIDCAB and OPCAB patients. Our findings indicate that there was no significant difference in all HRV measures between the two groups. However, results of our study should be interpreted with caution due to the relatively short data-recording time of 6 min.

Another surprising finding from our study was the significant improvement in pulmonary function after 6MWT in both MIDCAB and OPCAB patients. This suggests that walking can be beneficial for recovery following CABG surgery. Previous studies have confirmed that early exercise rehabilitation, including aerobic exercise such as walking, can effectively enhance cardiopulmonary function and exercise tolerance in patients after CABG [30]. However, we did not obtain preoperative pulmonary function data for these patients, which limited our ability to compare changes in pulmonary function before and after CABG surgery.

There are several limitations to this study. Firstly, as it is a retrospective cohort analysis, there may be biases in patient selection. Additionally, the sample size is limited due to the single-center nature of the study. Finally, each patient underwent the 6MWT only once after phase I CR due to constraints on time and staff. Further investigation is needed to confirm our findings in a larger, multi-center sample of MIDCAB patients to verify whether similar results are obtained in patients undergoing perioperative CR programs after other types of cardiothoracic surgery and to determine the long-term effects of rehabilitation.

## 5. Conclusions

In conclusion, MIDCAB had several advantages, including shorter surgery time, fewer grafted vessels, shorter ventilator-assisted time, fewer fluid infusion and bleeding, and shorter postoperative hospital stays. There was better 6MWT performance in MIDCAB patients following the perioperative CR program. Our findings support the use of the 6MWT as an assessment tool for patients prior to hospital discharge after CABG surgery. It can provide a feasible discharge planning for the phase II CR program of CABG patients, making it a valuable consideration for clinical application.

## Figures and Tables

**Table 1:** Clinical characteristics of participants between MIDCAB and OPCAB.

Variables	MIDCAB	OPCAB	Total	Statistics	*p*-Values
	n = 95 (31.2%)	n = 209 (68.8%)	n = 304 (100%)		
Age, y	63 ± 11	63 ± 13	63 ± 12	Z = −0.571	0.568 ^a^
Gender				χ^2^ = 4.289	**0.038 ^b^**
Male	81 (85.3%)	156 (74.6%)	237 (78.0%)		
Female	14 (14.7%)	53 (25.4%)	67 (22.0%)		
BMI, kg/m^2^	25.69 ± 2.93 *	25.88 ± 3.11 *	25.82 ± 3.05 *	t = −0.512	0.609 ^d^
Duration of symptoms, y	0.50 ± 1.92	1.00 ± 5.90	0.75 ± 4.92	Z = −1.495	0.135 ^a^
LAD, mm	41 ± 8	41 ± 8	41 ± 7	Z = −1.065	0.287 ^a^
LVEDD, mm	46 ± 7	47 ± 6	46 ± 6	Z = −0.816	0.414 ^a^
LVEF, %	62 ± 6	61 ± 7	62 ± 7	Z = −1.600	0.110 ^a^
Hypertension	62 (65.3%)	122 (58.4%)	184 (60.5%)	χ^2^ = 1.298	0.255 ^b^
Diabetes mellitus	39 (41.1%)	91 (43.5%)	130 (42.8%)	χ^2^ = 0.165	0.684 ^b^
Hyperlipidemia	49 (51.6%)	87 (41.6%)	136 (44.7%)	χ^2^ = 2.617	0.106 ^b^
Chronic lung disease	2 (2.1%)	11 (5.3%)	13 (4.3%)	χ^2^ = 0.913	0.339 ^c^
Cerebrovascular abnormality	17 (17.9%)	39 (18.7%)	56 (18.4%)	χ^2^ = 0.025	0.873 ^b^
Renal injury	3 (3.2%)	9 (4.3%)	12 (3.9%)	χ^2^ = 0.025	0.874 ^c^
Atrial fibrillation	0 (0.0%)	9 (4.3%)	9 (3.0%)	χ^2^ = 2.85	0.091 ^c^
Mitral insufficiency	7 (7.4%)	34 (16.3%)	41 (13.5%)	χ^2^ = 4.433	**0.035 ^b^**
Aortic insufficiency	3 (3.2%)	19 (9.1%)	22 (7.2%)	χ^2^ = 3.425	0.064 ^b^
Tricuspid insufficiency	11 (11.6%)	23 (11.0%)	34 (11.2%)	χ^2^ = 0.022	0.883 ^b^
Peripheral arterial disease	30 (31.6%)	87 (41.6%)	117 (38.5%)	χ^2^ = 2.785	0.095 ^b^
Prior myocardial infarction	2 (2.1%)	23 (11.0%)	25 (8.2%)	χ^2^ = 6.854	**0.009 ^b^**
Prior PCI	22 (23.2%)	47 (22.5%)	69 (22.7%)	χ^2^ = 0.017	0.897 ^b^
Smoking history	57 (60.0%)	125 (59.8%)	182 (59.9%)	χ^2^ = 0.001	0.975 ^b^
Drinking history	18 (18.9%)	39 (18.7%)	57 (18.8%)	χ^2^ = 0.004	0.953 ^b^
NYHA class				Z = −1.272	0.204 ^a^
Ⅱ	57 (60.0%)	109 (52.2%)	166 (54.6%)		
Ⅲ	38 (40.0%)	100 (47.8%)	138 (45.4%)		

MIDCAB, minimally invasive direct coronary artery bypass; OPCAB, off-pump coronary artery bypass; BMI, body mass index; LAD, left atrial diameter; LVEDD, left ventricular end-diastolic diameter; LVEF, left ventricular ejection fraction; PCI, percutaneous coronary intervention; NYHA, New York Heart Association. Results expressed as the median ± interquartile range (IQR) or number (%). * Mean and standard deviation. ^a^ Based on Mann–Whitney U test. ^b^ Based on chi-square test. ^c^ Based on chi-square test for continuity correction. ^d^ Based on independent Student’s *t*-test. Bold indicates a statistically significant difference.

**Table 2:** Surgical features of participants between MIDCAB and OPCAB.

Variables	MIDCAB	OPCAB	Total	Statistics	*p*-Values *
	n = 95 (31.2%)	n = 209 (68.8%)	n = 304 (100%)		
Total surgery time, h	4.03 ± 1.66	4.25 ± 1.33	4.17 ± 1.48	Z = −2.418	**0.016**
Number of grafted vessels, n	2 ± 2	3 ± 1	3 ± 1	Z = −7.821	**<0.001**
Ventilator-assisted time, h	4.18 ± 1.75	4.40 ± 2.29	4.33 ± 2.29	Z = −2.333	**0.020**
ICU length of stay, h	20.47 ± 6.90	19.27 ± 7.50	19.91 ± 7.24	Z = −0.644	0.520
Total intraoperative fluids, mL	2000 ± 750	2500 ± 1000	2250 ± 740	Z = −4.787	**<0.001**
Intraoperative bleeding, mL	400 ± 200	600 ± 300	600 ± 300	Z = −8.948	**<0.001**
Postoperative hospital stays, d	5 ± 1	6 ± 1	6 ± 2	Z = −5.379	**<0.001**

MIDCAB, minimally invasive direct coronary artery bypass; OPCAB, off-pump coronary artery bypass; ICU, intensive care unit. Results expressed as the median ± interquartile range (IQR) or number (%). * Based on Mann–Whitney U test. Bold indicates a statistically significant difference.

**Table 3:** Perioperative cardiac rehabilitation outcomes of participants between MIDCAB and OPCAB.

Variables	MIDCAB	OPCAB	Total	Statistics	*p*-Values *
	n= 95 (31.2%)	n = 209 (68.8%)	n = 304 (100%)		
6MWT					
Time from surgery to 6MWT, d	5 ± 2	5 ± 2	5 ± 2	Z = −3.501	**<0.001**
6MWD, m	200 ± 125	178 ± 125	182 ± 126	Z = −2.773	**0.006**
6MWD (% predicted), %	35 ± 23	33 ± 20	33 ± 21	Z = −2.506	**0.012**
METs, kcal/kg/h	2.70 ± 0.80	2.60 ± 0.80	2.60 ± 0.90	Z = −2.823	**0.005**
HRV during 6MWT					
NNVGR, ms	592.93 ± 104.11	579.33 ± 113.02	584.81 ± 111.08	Z = −1.468	0.142
SDNN, ms	23.20 ± 21.62	21.83 ± 16.88	22.11 ± 18.11	Z = −0.109	0.913
RMSSD, ms	22.12 ± 31.53	19.34 ± 26.31	19.90 ± 27.50	Z = −0.144	0.885
SDSD, ms	22.14 ± 31.57	19.35 ± 26.34	19.92 ± 27.52	Z = −0.144	0.885
NN50, n	3.00 ± 19.00	4.00 ± 13.00	4.00 ± 13.00	Z = −0.742	0.458
PNN50, %	0.51 ± 3.30	0.66 ± 1.96	0.62 ± 2.33	Z = −0.609	0.542
TP, ms^2^	177.36 ± 291.58	155.83 ± 272.76	159.57 ± 282.09	Z = −0.754	0.451
VLF, ms^2^	74.05 ± 95.39	76.75 ± 117.27	75.97 ± 106.14	Z = −0.011	0.991
LF, ms^2^	34.49 ± 54.58	26.02 ± 47.32	27.80 ± 47.95	Z = −1.544	0.123
HF, ms^2^	21.49 ± 135.08	26.36 ± 73.96	22.32 ± 83.51	Z = −0.316	0.752
LF/HF	1.12 ± 2.06	0.88 ± 1.35	0.95 ± 1.42	Z = −1.225	0.221
VLF/HF	3.03 ± 8.08	3.35 ± 6.91	3.14 ± 7.38	Z = −0.074	0.941
SD1, ms	16.25 ± 35.64	18.95 ± 28.49	17.83 ± 29.60	Z = −0.305	0.761
SD2, ms	27.66 ± 34.72	27.35 ± 29.14	27.39 ± 32.03	Z = −0.334	0.739
SD2/SD1	1.54 ± 0.57	1.43 ± 0.78	1.46 ± 0.74	Z = −1.404	0.160
UCG after surgery					
LAD, mm	36 ± 7	36 ± 9	36 ± 8	Z = −0.078	0.938
LVEDD, mm	44 ± 6	45 ± 7	45 ± 7	Z = −0.587	0.557
LVEF, %	60 ± 5	60 ± 7	60 ± 6	Z = −2.886	**0.004**

MIDCAB, minimally invasive direct coronary artery bypass; OPCAB, off-pump coronary artery bypass; 6MWT, 6 min walk test; 6MWD, 6 min walk distance; METs, metabolic equivalent; HRV, heart rate variability; NN, normal-to-normal RR intervals; NNVGR, normal-to-normal variability of the geometric range; SDNN, standard deviation of all NN intervals; RMSSD, root mean square of the difference between the coupling intervals of adjacent NN intervals; SDSD, standard deviation of successive differences; NN50, number of interval differences in successive NN intervals greater than 50 ms; PNN50, proportion of NN intervals differing by more than 50 ms; TP, total power; VLF, very low-frequency range; LF, low-frequency range; HF, high-frequency range; SD1, standard deviation perpendicular to the line-of-identity; SD2, standard deviation along the line-of-identity; UCG, ultrasonic cardiogram; LAD, left atrial diameter; LVEDD, left ventricular end-diastolic diameter; LVEF, left ventricular ejection fraction. Results are expressed as the median ± interquartile range (IQR) or number (%). * Based on Mann–Whitney U test. Bold indicates a statistically significant difference.

**Table 4:** Changes in lung function in MIDCAB and OPCAB group.

Variables	Groups	Time		Statistical Effect	F	*p*-Value *
		Before 6MWT (Median ± IQR)	After 6MWT (Median ± IQR)			
FVC (% predicted)	MIDCAB	38.00 ± 22.00	39.00 ± 14.00	Time × Groups	1.297	0.256
	OPCAB	39.00 ± 22.00	39.00 ± 19.00	Time	0.123	0.726
				Groups	0.046	0.830
FEV1 (% predicted)	MIDCAB	35.00 ± 13.00	39.00 ± 16.00	Time × Groups	0.768	0.382
	OPCAB	35.00 ± 13.50	38.00 ± 18.00	Time	14.503	**<** **0.001**
				Groups	0.046	0.831
PEF (% predicted)	MIDCAB	20.00 ± 17.00	23.00 ± 18.00	Time × Groups	0.148	0.701
	OPCAB	21.00 ± 15.00	22.00 ± 17.00	Time	4.562	**0.033**
				Groups	0.792	0.374
FEV1/FVC (% predicted)	MIDCAB	81.00 ± 29.00	83.00 ± 20.00	Time × Groups	<0.001	0.986
	OPCAB	81.00 ± 31.50	85.00 ± 23.50	Time	14.762	**<** **0.001**
				Groups	0.147	0.702
FEF25 (% predicted)	MIDCAB	17.00 ± 12.00	20.00 ± 16.00	Time × Groups	0.004	0.948
	OPCAB	17.00 ± 13.00	20.00 ± 16.00	Time	21.601	**<** **0.001**
				Groups	0.456	0.500
FEF50 (% predicted)	MIDCAB	27.00 ± 15.00	32.00 ± 18.00	Time × Groups	0.075	0.785
	OPCAB	27.00 ± 17.00	30.00 ± 20.50	Time	24.366	**<** **0.001**
				Groups	0.318	0.573
FEF75 (% predicted)	MIDCAB	64.00 ± 37.00	79.00 ± 45.00	Time × Groups	0.879	0.349
	OPCAB	65.00 ± 52.50	71.00 ± 66.00	Time	7.784	**0.006**
				Groups	0.430	0.512
FEF25–75 (% predicted)	MIDCAB	33.00 ± 12.00	34.00 ± 15.00	Time × Groups	0.168	0.682
	OPCAB	32.00 ± 12.50	34.00 ± 16.00	Time	13.992	**<** **0.001**
				Groups	0.140	0.708

MIDCAB, minimally invasive direct coronary artery bypass; OPCAB, off-pump coronary artery bypass; 6MWT, 6 min walk test; FVC, forced vital capacity; FEV1, forced expiratory volume in one second; PEF, peak expiratory flow; FEF, forced expiratory flow; IQR, interquartile range. * Based on Repeated-Measures ANOVA. Bold indicates a statistically significant difference.

## Data Availability

The original contributions presented in this study are included in the article. Further inquiries can be directed to the corresponding author.
